# Statin use and risk of contralateral breast cancer: a nationwide cohort study

**DOI:** 10.1038/s41416-018-0252-1

**Published:** 2018-10-24

**Authors:** Rikke Langballe, Deirdre Cronin-Fenton, Christian Dehlendorff, Maj-Britt Jensen, Bent Ejlertsen, Michael Andersson, Søren Friis, Lene Mellemkjær

**Affiliations:** 1 Danish Cancer Society Research Center, Copenhagen, Denmark; 2 Department of Clinical Epidemiology, Aarhus University Hospital, Aarhus, Denmark; 3 Danish Breast Cancer Group, Rigshospitalet, Copenhagen, Denmark; 4 Department of Oncology, Rigshospitalet, Copenhagen, Denmark; 5 Department of Public Health, Copenhagen University, Copenhagen, Denmark

## Abstract

**Background:**

Statins have demonstrated antineoplastic effects in breast cancer cell lines, particularly in oestrogen receptor (ER)-negative cell lines. However, epidemiological studies have not supported a preventive effect of statin use against breast cancer. Therefore, we examined the association between statin use and contralateral breast cancer (CBC) risk among women with breast cancer.

**Methods:**

We identified 52,723 women with non-metastatic breast cancer during 1996–2012 from the Danish Breast Cancer Group database. We defined time-varying post-diagnosis statin use as minimum two prescriptions lagged by 1 year. Cox regression analyses were used to estimate hazard ratios (HRs) and 95% confidence intervals (CIs) for CBC associated with statin use.

**Results:**

Statin use was associated with a lower CBC risk (HR = 0.88; 95% CI = 0.73–1.05). The inverse association was strongest for long-term use overall (HR = 0.64; 95% CI = 0.43–0.96), although the HR specifically for long-term consistent use and high-intensity use approached unity. Among ER-negative breast cancer patients, statin use was associated with a CBC risk reduction (HR = 0.67; 95% CI = 0.45–1.00).

**Conclusions:**

We found some indication that statins reduce the risk of CBC. Further evaluations are needed to disentangle the equivocal results for long-term use and to establish if ER-negative breast cancer patients may benefit most from statin use.

## Introduction

Statins reduce serum levels of cholesterol and prevent cardiovascular disease.^1^ Beneficial effects of statins against breast cancer have also been suggested in studies of breast cancer cell lines and animal models.^2,3^ These antineoplastic effects may be explained through blocking of the mevalonate pathway responsible for the synthesis of various products including cholesterol and isoprenoids that are involved in tumour initiation, growth and metastasis.^4^ Experimental studies have also suggested that the anti-neoplastic effects are limited to lipophilic statins, and notably pertain to oestrogen receptor (ER)-negative breast tumours, whereas hydrophilic statins have demonstrated no anti-neoplastic effects, regardless of ER status.^2,5^ Although the experimental findings are promising, epidemiologic studies of statin use and breast cancer risk have reported largely null associations, and a recent meta-analysis of 27 epidemiological studies and 9 randomised clinical trials (RCTs) concluded that statin use does not reduce the risk of breast cancer.^6^

A potential explanation of the null findings in studies of statin use and breast cancer risk may be that preventive effects of statins are attenuated in the general female population who have varying degrees of susceptibility to breast cancer. However, women who have developed breast cancer have proven susceptibility to the disease, and preventive measures against breast cancer may be more pronounced in such a high risk population. A study based on the Danish Breast Cancer Group (DBCG) and the nationwide Danish Prescription Registry reported a 46% reduced risk of contralateral breast cancer (CBC) associated with use of simvastatin, a lipophilic statin, up to 10 years after primary breast cancer diagnosis.^7^ However, CBC was not the primary outcome in that study, and no details on the association with CBC risk were presented.

We hypothesise that statin use reduces CBC risk among women with breast cancer, and that the protective effect is most pronounced in women with an ER-negative first breast cancer.

## Methods

### Study population

We identified a cohort of patients with incident invasive breast cancer within the DBCG database.^8^ The DBCG database became nationwide in 1977 and includes detailed clinical information on the vast majority of patients (see Supplemental Material, Box 1 for additional details on the DBCG database and other nationwide registries used in the study).

**Fig. 1:**
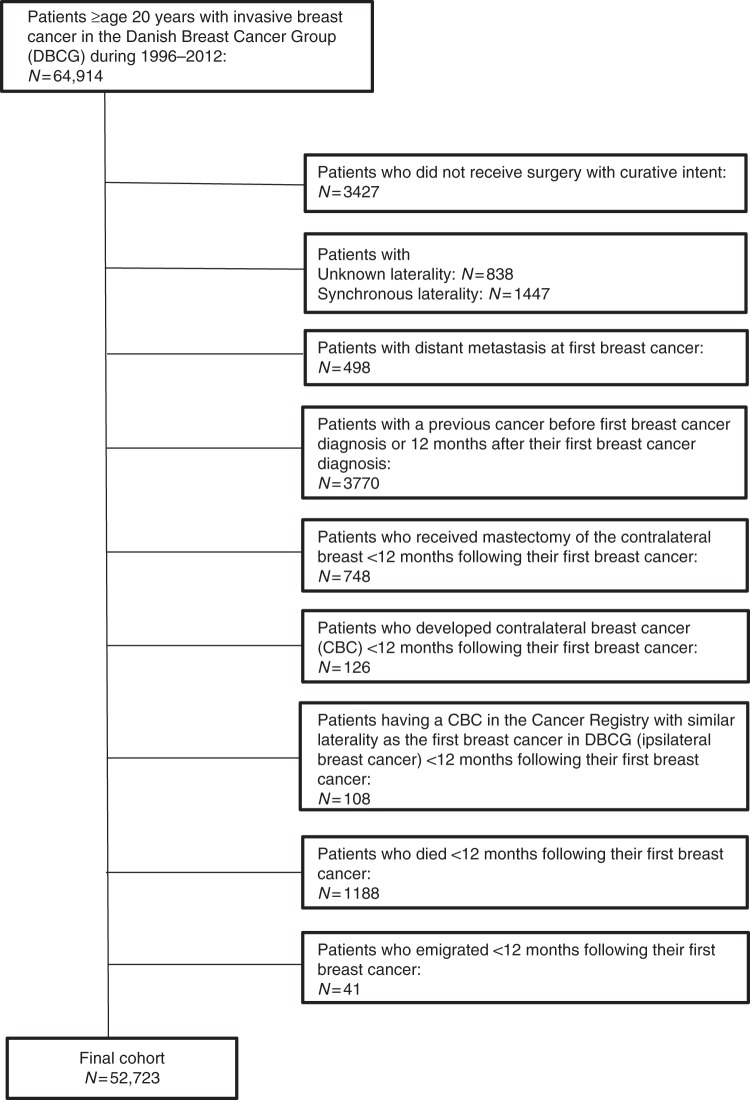
Flow chart of study participants

The cohort comprised 64,914 women aged ≥20 years with a first primary invasive breast cancer during 1996–2012. By starting the study cohort in 1996, we had at least one year of pre-diagnosis statin use as the National Prescription Registry was first initiated in 1995.^9^ We excluded patients who did not undergo surgery with curative intent (*N* = 3427), had no record of laterality of the initial breast cancer (*N* = 838), or had synchronous (*N* = 1 447) or metastatic disease (*N* = 498) at diagnosis. In addition, by linkage to the Danish Cancer Registry,^10^ we excluded patients who had a history of cancer (except non-melanoma skin cancer) prior to or within 1 year after the primary breast cancer (*N* = 3770). Finally, we excluded women who received mastectomy of the contralateral breast (*N* = 748) or developed cancer in the contralateral or same breast within the first year after the first breast cancer diagnosis (*N* = 234), and women who died or emigrated during this period (*N* = 1229). The final cohort comprised 52,723 breast cancer patients who were alive and at risk of CBC 1 year after the diagnosis (see flow chart in Fig. 1).

### Assessment of statin use

From the National Prescription Registry,^9^ we retrieved information on statin prescriptions filled by the breast cancer patients between 1995 and 2013. We defined post-diagnosis statin use (see Supplemental Material, Table 1) as at least two prescriptions recorded on separate dates after the primary breast cancer diagnosis. Post-diagnosis statin use was lagged 1 year to allow a meaningful biological latency and to avoid reverse causation.^11,12^

**Table 1:** Baseline characteristics of 52,723 patients diagnosed with a first primary stage I–III breast cancer in Denmark during 1996–2012 according to statin use within the first year after cancer diagnosis

Characteristics	Statin use within the year following the first breast cancer^a^	
	Yes	No
	Number (%)	Number (%)
**All**	5481 (100)	47,242 (100)
Age at first breast cancer diagnosis (years)^b^		
20–49	133 (2)	10,454 (22)
50–59	991 (18)	13,457 (28)
60–64	1146 (21)	7078 (15)
65–69	1351 (25)	5997 (13)
70–79	1404 (26)	6854 (15)
>=80	456 (8)	3402 (7)
Menopausal status at first breast cancer diagnosis^b^		
Premenopausal	239 (4)	13,108 (28)
Postmenopausal	5118 (94)	33,548 (71)
Unknown	124 (2)	586 (1)
Calendar period of first breast cancer^b^		
1996–2000	235 (4)	13,416 (28)
2001–2004	651 (12)	10,944 (23)
2005–2008	1709 (31)	10,634 (23)
2009–2012	2886 (53)	12,248 (26)
Histology of first breast cancer^b^		
Ductal	4299 (78)	37,577 (79)
Lobular	533 (10)	5147 (11)
Other	618 (11)	4194 (9)
Unknown	31 (1)	324 (1)
Histology and grade of first breast cancer^b^		
Ductal, Grade I	1397 (25)	11,288 (24)
Ductal, Grade II–III	2801 (51)	25,337 (53)
Other, Grade I–III/unknown	1151 (21)	9341 (20)
Unknown	132 (2)	1276 (3)
ER status at first breast cancer^b^		
ER positive	4565 (83)	37,028 (78)
ER negative	844 (15)	8980 (19)
Unknown	72 (1)	1234 (3)
Tumour size of first breast cancer^b^		
<=2 cm	3413 (62)	27,667 (59)
2.1–5 cm	1834 (34)	16,726 (35)
>=5.1 cm	103 (2)	1618 (3)
Unknown	131 (2)	1231 (3)
Lymph node status of first breast cancer^b^		
Negative	3110 (57)	24,359 (52)
Positive	2166 (39)	20,932 (44)
Unknown	205 (4)	1951 (4)
Treatment for first breast cancer^c^		
No treatment	278 (5)	4189 (9)
Radiation treatment only	822 (15)	6173 (13)
Chemotherapy only	199 (4)	1988 (4)
Chemotherapy+radiation treatment	466 (9)	4994 (10)
Endocrine treatment only	784 (14)	4736 (10)
Endocrine treatment+radiation treatment	1967 (35)	9744 (21)
Endocrine treatment+chemotherapy	80 (2)	1360 (3)
Endocrine treatment+chemotherapy+ radiation treatment	518 (10)	7905 (17)
Unknown treatment	367 (6)	6153 (13)
Comorbidity^d^		
Tobacco related diseases	437 (8)	2320 (5)
Alcohol related diseases	101 (2)	748 (2)
Diabetes	928 (17)	1047 (2)
Other drug exposure		
HRT, pre-diagnosis^e^	1344 (25)	11,422 (24)
Aspirin, one-year post-diagnosis^a^	2133 (39)	3242 (7)
Bisphosphonates, one-year post-diagnosis^a^	354 (7)	1418 (3)
Metformin, one-year post-diagnosis^a^	785 (14)	487 (1)
Digoxin, one-year post-diagnosis^a^	180 (3)	923 (2)
Educational level at first breast cancer diagnosis		
Short (up to 9 years)	1853 (34)	11,209 (24)
Medium (10–12 years)	2632 (48)	21,296 (45)
High (>12 years)	864 (16)	11,526 (24)
Unknown	132 (2)	3211 (7)

*CBC* contralateral breast cancer, *ER* oestrogen receptor status, *HRT* hormone replacement therapy

^a^Defined as ≥2 prescriptions during the first year after the first breast cancer diagnosis

^b^Information retrieved from the Danish Breast Cancer Group

^c^Intention to treat treatment retrieved from the Danish Breast Cancer Group

^d^Defined as tobacco-related diseases, alcohol-related diseases and diabetes mellitus 10 years prior to or one year after first breast cancer diagnosis

^e^Defined as ≥2 prescriptions within one year prior to the first breast cancer diagnosis

In the main analysis, post-diagnosis statin use was modelled as a time-dependent variable. Thus, exposed person-time began from 1 year after the second prescription of statins, and person-time up to this point was considered as non-use. Moreover, we defined current statin use consecutively throughout follow-up as the period from filling a prescription plus the number of tablets and additional 90 days forward to allow minor non-compliance. Past-use of statins was defined as person-time among users not fulfilling the definition of exposure period for current use. Statin users were classified as consistent users until past use, if any, occurred where after they were classified as irregular users. Duration of statin use was defined as the interval between the first and latest prescription plus the number of tablets in the latest prescription and categorised as <5 years or ≥5 years. Intensity of statin use (average daily statin dose) was consecutively estimated during follow-up as the cumulative number of DDDs divided by the number of days between the first and the latest statin prescription and classified as <1 DDD/day or >=1 DDD/day. Finally, we categorised subtypes of statins according to solubility (lipophilic/hydrophilic, see Table 2).

**Table 2:** Hazard ratios (HRs) and 95% confidence intervals (CIs) of contralateral breast cancer (CBC) associated with post-diagnosis statin use, patterns of statin use and type of statin among 52,723 breast cancer patients during 1996–2012 in Denmark

Post-diagnosis statin use	Person-years	*N*	Number of CBCs	Age-adjusted model		Fully adjusted model^a^	
				HR	95% CI	HR	95% CI
Non-use	266,790	52,723	1204	1	Reference	1	Reference
Ever use^b^	43,747	11,507	178	0.84	0.71–0.99	0.88	0.73–1.05
Current or past use^c^							
Non-use	266,790	52,723	1204	1	Reference	1	Reference
Current use	38,932	11,507	156	0.83	0.70–0.99	0.87	0.72–1.04
Past use	4815	4502	22	0.91	0.60–1.40	0.95	0.62–1.46
Duration^d^ and intensity of use^e^							
Non-use	266,790	52,723	1 204	1	Reference	1	Reference
<5 years	36,145	11,488	152	0.89	0.75–1.05	0.93	0.77–1.11
1 DDD/day	19,262	6896	81	0.88	0.70–1.11	0.90	0.71–1.14
>=1 DDD/day	16,884	6952	71	0.89	0.70–1.13	0.96	0.75–1.24
>5 years	7602	3121	26	0.63	0.42–0.94	0.64	0.43–0.96
<1 DDD/day	4763	1920	12	0.46	0.26–0.82	0.46	0.26–0.83
>=1 DDD/day	2839	1422	14	0.92	0.54–1.56	0.95	0.55–1.64
Consistency of use^f^ and duration^d^							
Non-use	266 790	52 723	1 204	1	Reference	1	Reference
Consistent	30 920	11 507	134	0.91	0.75–1.09	0.95	0.78–1.15
<5 years	27 000	11 488	114	0.90	0.74–1.09	0.95	0.77–1.16
>=5 years	3 920	1 809	20	0.94	0.60–1.47	0.96	0.61–1.52
Irregular	12 827	4 502	44	0.68	0.50–0.93	0.71	0.51–0.97
Type of statin							
Non-use	266 790	52 723	1 204	1	Reference	1	Reference
Lipophilic statins^g^	39 093	10 823	157	0.83	0.70–0.98	0.87	0.72–1.04
Only simvastatin	32 812	9 810	127	0.80	0.66–0.97	0.85	0.69–1.04
Only atorvastatin	2 552	869	10	0.84	0.45–1.58	0.82	0.44–1.54
Other lipophilic statins^h^	3 729	1 100	20	1.07	0.68–1.67	1.06	0.67–1.67
Hydrophilic statins^i^	1 723	684	11	1.35	0.75–2.45	1.37	0.75–2.49
Mixed use^j^	2 931	935	10	0.67	0.36–1.26	0.70	0.37–1.31

^a^Adjusted for age at first breast cancer, calendar-period at first breast cancer (1996–2000/2001–2004/2005–2008/2009–2012), lobular histology of first breast cancer (yes/no), treatment for first breast cancer (endocrine treatment only, chemotherapy only, radiation treatment only, endocrine treatment+chemotherapy, endocrine treatment+radiation treatment, chemotherapy+radiation treatment, endocrine treatment+chemotherapy+radiation treatment, no treatment and unknown treatment), pre-diagnosis exposure to hormone-replacement therapy (yes/no), time-dependent post-diagnosis exposure to aspirin, bisphosphonates, metformin and digoxin, alcohol-related conditions (yes/no), tobacco-related conditions (yes/no), diabetes mellitus (yes/no), educational level at first breast cancer diagnosis (short, medium, high, unknown)

^b^Ever statin use was defined as >=2 prescriptions after the first breast cancer diagnosis

^c^Current use was defined as the period from redeeming a prescription plus the number of tablets and additional 90 days forward. Past use was defined as person-time among users not defined as current use

^d^Duration of statin use was defined as the interval between the first and latest prescription plus the number of tablets in the latest prescription

^e^Intensity of use was defined as the cumulative number of defined daily doses (DDD) divided by duration of use in days

^f^Consistency of use was defined as current users with no prior periods as past users

^g^Lipophilic statin use was defined as users of simvastatin, atorvastatin, lovastatin, fluvastatin and cerivastatin only

^h^Other lipophilic statin use was defined as users of lovastatin, fluvastatin and cerivastatin only

^i^Hydrophilic statins was defined as users of pravastatin and rosuvastatin only

^j^Mixed use was defined as more than one type, e.g. simvastatin and atorvastatin

### Follow-up for contralateral breast cancer

We used a previously established nationwide database on CBCs derived from the Danish Cancer Registry.^10^ According to coding rules in the Cancer Registry,^10^ a second primary cancer occurring in paired organs with similar histology as the first primary tumour is not registered as an individual cancer record, but only labelled as ‘bilateral’. Therefore, we obtained additional information on the CBC from the original notification forms to the Cancer Registry during 1978–2003, and from electronic records in the Danish Pathology Register during 2004–2013.^13^ Additional CBCs recorded in the DBCG only were added to the database. CBC with distant metastases at diagnosis was not counted as an outcome to limit possible misclassification of metastases from the first breast cancer (*N* = 58), but used as a censoring variable.

Follow-up for CBC started 1 year after the first breast cancer diagnosis (baseline) and continued until CBC, other cancer diagnosis (except non-melanoma skin cancer), ipsilateral breast cancer, prophylactic mastectomy of the contralateral breast, death, emigration or end of 2013, whichever came first. Contralateral mastectomy (see Supplemental Material, Table 1) was retrieved from the Danish National Patient Register.^14^ We lagged the date of mastectomy by 30 days to ensure the procedure was prophylactic. Information on death and emigration was obtained from the Danish Civil Registration System.^15^

### Definition of covariates

Information on potential confounding factors was retrieved from data available in the nationwide registries in Denmark. From DBCG, we included information on the primary breast cancer diagnosis: age (through a restricted cubic spline), date of breast cancer diagnosis (1996–2000/2001–2004/2005–2008/2009–2012), lobular histology (yes/no), and treatment (in eight categories; see footnote in Table 2). The DBCG guidelines define standard treatment modalities, which patients are allocated to according to age at diagnosis and tumour characteristics.^16^ We used the allocated treatment as adjustment variable in the analyses. Information on diabetes and alcohol- or tobacco-related diseases (yes/no) from 10 years prior to 1 year after the primary breast cancer was retrieved from the Danish National Patient Register.^14^ We also obtained information on use of prescription drugs from the National Prescription Registry, including pre-diagnosis hormone replacement therapy (>2 prescriptions) and post-diagnosis use (>2 prescriptions) of aspirin, bisphosphonates, metformin and digoxin. The Supplemental Material provides a detailed description of codes for drug exposure and covariates. From Statistics Denmark,^17^ we retrieved information on the highest achieved educational level at primary breast cancer diagnosis (short: up to 9 years; medium: 10–12 years; and high: >12 years).

### Statistical analyses

We used cause-specific Cox regression models to estimate age- and fully adjusted hazard ratios (HRs) and 95% confidence intervals (CIs) for risk of CBC associated with statin use. The fully adjusted model included the covariates defined above and the presented results in the text are from these models. Time since baseline was used as the underlying time scale. In the main analyses, we evaluated the association between post-diagnosis use of statins and patterns of statin use (current/past-use, duration, intensity and consistency) and type of statins as time-varying categorical variables compared to non-use. Effect measure modification was evaluated by stratified analyses according to information from DBCG on ER status of the primary breast cancer. Moreover, we analysed risk of ER-positive and ER-negative CBC associated with statin use by censoring at date of a non ER-positive and non ER-negative CBC, respectively. We repeated the main analyses restricting statin exposure to (1) lipophilic statins only or (2) simvastatin only (see Supplemental Material for ATC codes). The proportional hazards assumption was evaluated by testing for trends in the scaled Schoenfeld residuals for ever use vs. non-use in the fully adjusted model.

In secondary analyses, we evaluated the influence of statin use prior to the first breast cancer on risk of CBC, by using a time-varying exposure matrix combining pre- and post-diagnosis statin use, i.e., (1) no use before or after first breast cancer diagnosis (reference), (2) use before the diagnosis, (3) use solely after the first breast cancer diagnosis, and (4) use both before and after the diagnosis. This analysis was restricted to women diagnosed between 1997 and 2012 to allow a pre-diagnosis exposure window of at least 2 years.

We performed two pre-defined sensitivity analyses. First, we changed the definition of statin use to one prescription in the main analyses. Second, we examined the association between statin use (≥2 prescriptions) and CBC risk using a fixed exposure period of 1 year following the first breast cancer diagnosis.

In post hoc sensitivity analyses, we also adjusted for menopausal status (premenopausal/postmenopausal) at first breast cancer diagnosis (women with unknown menopausal status (*N* = 710) were classified as premenopausal if younger than 50 years at diagnosis and postmenopausal if older than 50 years at diagnosis). In addition, we calculated a yearly updated propensity score using a logistic regression model to estimate the probability of being statin user during follow-up (time-varying) or the first year after breast cancer diagnosis (time-fixed). In the propensity model we included the same covariates as in the fully adjusted model as well as menopausal status, lymph node status (positive/negative/unknown) and ER status (positive/negative/unknown) at first breast cancer diagnosis.^18^ In the Cox model, the propensity score was included through a restricted cubic spline together with age at first breast cancer and statin use (ever use/non-use).

Finally, we evaluated the potential impact of competing events in all analyses by estimating age- and fully adjusted HRs for the censoring criteria as one combined outcome (death, other cancer, ipsilateral breast cancer, distant disease at CBC diagnosis and mastectomy of the contralateral breast).

All analyses were performed in R version 3.2.3^19^ using the survival package,^20^ applying a 5% significance level and two-sided alternatives.

## Results

During the first year following the primary breast cancer, 5481 patients filled two or more statin prescriptions (Table 1). Statin users were more likely to be postmenopausal at first breast cancer diagnosis than non-users. The prevalence of statin use was higher among breast cancer patients in the later part of the study period, reflecting the increasing use of statins in the general population.^21^ Furthermore, statin users had a higher prevalence of diabetes, aspirin and metformin use, and shorter education compared with non-users.

During 310,537 person-years, 1382 breast cancer patients developed CBC. Overall, 11,507 patients filled two or more statin prescriptions and contributed person-years as statin users for a median of 3.3 years (interquartile range: 1.6–5.4 years). Post-diagnosis statin use was associated with a slightly reduced risk of CBC (HR = 0.88; 95% CI = 0.73–1.05) (Table 2). A similar HR was seen with current statin use (HR = 0.87; 95% CI = 0.72–1.04), whereas the HR for CBC with past-use of statins approached unity (HR = 0.95; 95% CI = 0.62–1.46), however, numbers were small for past statin use.

We observed a reduced risk for CBC (HR = 0.64; 95% CI = 0.43–0.96) associated with post-diagnosis long-term use (>5 years), whereas the association with shorter duration of statin use was close to unity (<5 years) (HR = 0.93; 95% CI = 0.77–1.11) (Table 2). Stratification of duration of use according to intensity yielded a substantially reduced risk of CBC associated with long-term (>5 years), low intensity ( < 1 DDD/day) statin use (HR = 0.46; 95% CI = 0.26–0.83), whereas no apparent inverse association emerged for long-term, high-intensity (>=1 DDD/day) statin use (HR = 0.95; 95% CI = 0.55–1.64). In addition, we observed a reduced risk for CBC for irregular use of statins (HR = 0.71; 95% CI = 0.51–0.97), but not for consistent use (HR = 0.95; 95% CI = 0.78–1.15). In a post hoc analysis, we found no difference in CBC risk when consistent statin use was stratified into short-term use (<5 years: HR = 0.95; 95% CI = 0.77–1.16) and long-term use (>5 years: HR = 0.96; 95% CI = 0.61–1.52). The majority of patients used the highly lipophilic statin, simvastatin (71%) and a slightly reduced risk of CBC was seen for these users (HR = 0.85; 95% CI = 0.69–1.04). Use of hydrophilic statins was associated with a statistically insignificant increased risk of CBC (HR: 1.37, 95% CI = 0.75–2.49), however, use of these agents were rare and the number of CBC was small. When restricting the analyses on patterns of use to lipophilic statins or simvastatin alone, the HRs remained unaltered (data not shown).

Among women with ER-negative primary breast cancer, we observed a reduced risk of CBC (HR = 0.67; 95% CI = 0.45–1.00) associated with ever post-diagnosis statin use (Table 3), whereas no apparent inverse association was seen among women with ER-positive tumours (HR = 0.94; 95% CI = 0.77–1.14). Among the statin users with ER-negative initial breast cancer, the HRs for ER-positive and ER-negative CBC were 0.64 (95% CI = 0.38–1.08) and 0.77 (95% CI = 0.30–1.95), respectively. Restriction of these analyses to lipophilic use only yielded HRs similar to those in the main analyses (Supplemental Material, Table 2).

**Table 3:** Hazard ratios (HRs) of contralateral breast cancer (CBC) and 95% confidence intervals (CIs) associated with post-diagnosis statin use according to oestrogen receptor (ER) status of first breast cancer (BC) diagnosis and CBC among 52,723 breast cancer patients during 1996–2012 in Denmark

	Person-years	*N*	Number of CBCs	Age-adjusted model		Fully adjusted model^a^	
				HR	95% CI	HR	95% CI
*ER status of first breast cancer*							
Positive							
Non-use	208,219	41,593	885	1	Reference	1	Reference
Ever statin use	34,797	9331	144	0.89	0.74–1.06	0.94	0.77–1.14
Negative							
Non-use	49,257	9824	274	1	Reference	1	Reference
Ever statin use	7677	1903	28	0.65	0.44–0.97	0.67	0.45–1.00
Unknown							
Non-use	9313	1306	45	1	Reference	1	Reference
Ever statin use	1273	273	6	0.16	0.06–0.38	0.17	0.07–0.40
*ER status of contralateral breast cancer* ^b^							
Positive^c^							
Non-use	266,790	52,723	818	1	Reference	1	Reference
Ever statin use	43,747	11,507	136	0.86	0.71–1.04	0.86	0.70–1.06
Negative^d^							
Non-use	266,790	52,723	214	1	Reference	1	Reference
Ever statin use	43,747	11,507	26	0.91	0.59–1.38	0.88	0.55–1.41
*ER status of first and second breast cancer*							
ER-positive first BC and ER-positive CBC^c^							
Non-use	208,219	41,593	652	1	Reference	1	Reference
Ever statin use	34,797	9331	112	0.89	0.73–1.10	0.93	0.74–1.16
ER-positive first BC and ER-negative CBC^d^							
Non-user	208,219	41,593	120	1	Reference	1	Reference
Ever statin use	34,797	9331	18	0.90	0.54–1.49	0.91	0.51–1.61
ER-negative first BC and ER-positive CBC^c^							
Non-use	49,257	9824	142	1	Reference	1	Reference
Ever statin use	7677	1903	21	0.73	0.46–1.17	0.64	0.38–1.08
ER-negative first BC and ER-negative CBC^d^							
Non-use	49,257	9824	86	1	Reference	1	Reference
Ever statin use	7677	1903	6	0.73	0.31–1.71	0.77	0.30–1.95

^a^Adjusted for age at first breast cancer, calendar-period at first breast cancer (1996–2000/2001–2004/2005–2008/2009–2012), lobular histology of first breast cancer (yes/no), treatment for first breast cancer (endocrine treatment only, chemotherapy only, radiation treatment only, endocrine treatment+chemotherapy, endocrine treatment+radiation treatment, chemotherapy+radiation treatment, endocrine treatment+chemotherapy+radiation treatment, no treatment and unknown treatment), pre-diagnosis exposure to hormone-replacement therapy (yes/no), time-dependent post-diagnosis exposure to aspirin, bisphosphonates, metformin and digoxin, alcohol-related conditions (yes/no), tobacco-related conditions (yes/no), diabetes mellitus (yes/no) and educational level at first breast cancer diagnosis (short, medium, higher, unknown)

^b^We have not estimated HR for unknown ER status of the CBC

^c^In these sub analyses, ER-positive contralateral breast cancer was the outcome of interest and ER-negative and unknown ER status was censuring variables plus all the censuring variables used in all other analyses

^d^In these sub analyses, ER-negative contralateral breast cancer was the outcome of interest and ER-positive and unknown ER status was censuring variables plus all the censuring variables used in all other analyses

Among patients diagnosed between 1997 and 2012, pre-diagnosis statin use was associated with a neutral HR for CBC compared with never-use (HR = 0.97; 95% CI = 0.53–1.80) (Table 4), whereas slightly reduced HRs were observed for new post-diagnosis users (HR = 0.89; 95% CI = 0.71–1.10) and for continuing users (HR = 0.84; 95% CI = 0.62–1.12).

**Table 4:** Hazard ratios (HRs) of contralateral breast cancer (CBC) and 95% confidence intervals (CIs) associated with timing of statin use among 50,087 breast cancer patients during 1997–2012^a^ in Denmark

Timing of statin use	Person-years	*N*	Number of CBCs	Age adjusted model		Fully adjusted model^b^	
				HR	95% CI	HR	95% CI
Never-use^c^	239,644	44,692	1063	1	Reference	1	Reference
Pre-diagnosis use^d^	3363	5395	11	0.95	0.52–1.75	0.97	0.53–1.80
Post-diagnosis use only (‘new users’)	24,957	6339	107	0.85	0.69–1.05	0.89	0.71–1.10
Post- & pre-diagnosis use^e^ (‘continuers’)^e^	16,050	4691	56	0.79	0.60–1.04	0.84	0.62–1.12

^a^This analysis was restricted to women diagnosed during 1997–2012 to allow pre-diagnosis exposure window of at least two years (>2 prescriptions)

^b^Adjusted for age at first breast cancer, calendar-period at first breast cancer (1996–2000/2001–2004/2005–2008/2009–2012), lobular histology of first breast cancer (yes/no), treatment for first breast cancer (endocrine treatment only, chemotherapy only, radiation treatment only, endocrine treatment+chemotherapy, endocrine treatment+radiation treatment, chemotherapy+radiation treatment, endocrine treatment+chemotherapy+radiation treatment, no treatment and unknown treatment), pre-diagnosis exposure to hormone-replacement therapy (yes/no), time-dependent post-diagnosis exposure to aspirin, bisphosphonates, metformin and digoxin, alcohol-related conditions (yes/no), tobacco-related conditions (yes/no), diabetes mellitus (yes/no), educational level at first breast cancer diagnosis (short, medium, higher, unknown)

^c^Differs from the non-user category in Tables 2, 3 by not including person-years among pre-diagnosis users

^d^Defined as ≥2 prescriptions within 2 years prior to the first breast cancer diagnosis

^e^Defined as post-diagnosis users also having ≥2 prescriptions within 2 years prior to the first breast cancer diagnosis counting person-years from the 2nd prescription post-diagnosis lagged by 1 year

The sensitivity analysis defining statin use as ≥1 prescription showed no major change in the HR for overall post-diagnosis statin use (HR = 0.84; 95% CI = 0.71–1.00). The analysis applying a fixed exposure period within the first year following the primary breast cancer yielded a HR of 0.76 (95% CI = 0.58–1.00).

In post hoc sensitivity analyses, we found no change in the risk estimates when adding menopausal status at first breast cancer diagnosis as a confounder. Finally, adjusting for the propensity of being statin user yielded virtually the same results as in the fully adjusted models.

The analyses for competing events showed largely the same pattern as for CBC risk estimates, i.e., use of statins was associated with a reduced risk of the competing events.

## Discussion

In this population-based cohort study of 52,723 breast cancer patients, we found a tendency towards a slightly reduced risk of CBC associated with ever and current statin use and a substantial reduction in CBC risk with long-term statin use. Somewhat puzzling, we observed CBC risk reductions associated with long-term statin use of low intensity or irregular use, but not with long-term high intensity or consistent statin use. A substantial reduction in CBC risk with statin use was seen among patients with ER-negative primary breast cancer.

A Danish cohort study^7^ of 18,769 breast cancer patients from DBCG diagnosed during 1996–2003, thus overlapping with our study population, reported a reduction in CBC risk associated with the most commonly used lipophilic statin, simvastatin (HR = 0.54; 95% CI = 0.33–0.90). That association was based on 520 women with CBC within 10 years of their first breast cancer. A more recent, but smaller, cohort study from the United States^22^ had insufficient power to allow a meaningful conclusion on CBC risk. Our study was larger than previous studies and had more detailed information on statin type, timing and patterns of statin use, as well as on ER status of breast tumours.

Lipophilic statins have been suggested to possess the strongest antineoplastic effects due to their ability to cross cell membranes.^2^ Similar to our study, a meta-analysis of 10 studies evaluating breast cancer risk according to drug solubility has reported slight risk reductions associated with the highly lipophilic statin, simvastatin.^6^ Thus, so far there is a suggestion from this meta-analysis and the previous Danish study^7^ as well as our study that simvastatin use is associated with a decreased breast cancer risk.

Long-term use of statins (>5 years) was suggested to be associated with a reduced risk of breast cancer in a meta-analysis based on 8 studies.^6^ This previous study is compatible with our finding of a reduced CBC risk associated with long-term use for more than 5 years. However, when we combined long-term use with the highest level of daily dose or with use without exposure gaps to identify the women who would be most heavily exposed to statins, we did not observe a pattern supporting a duration- or dose-response relationship. However, the results were based on relatively small numbers. Additional studies are needed to explore these equivocalities in our results, e.g. to investigate the dose level required to achieve antineoplastic effects of statins in humans.

Preventive measures for ER-negative breast cancer are highly warranted.^23,24^ Besides, women with ER-negative breast cancer are at increased risk of CBC compared with women diagnosed with the ER-positive phenotype, and in particular ER-negative CBC.^25,26^ Previous epidemiological studies of either lipophilic statin use specifically^27,28^ or any statin type^29–31^ have found no evidence of a differential effect by ER status. Our study suggested a reduced risk of CBC associated with statin use in patients with ER-negative primary breast cancer. This finding could imply an additional benefit of post-diagnosis statin use in addition to the growing evidence of reduced mortality^32–35^ and reduced risk of recurrences^3,7,34,36^ associated with statin use *after* breast cancer. Therefore, our results deserve to be evaluated further.

The strengths of our study included comprehensive nationwide data from a clinical database (DBCG), ensuring an unselected study population of breast cancer patients with high case validity. This was supplemented by high quality data from nationwide demographic and health registries ensuring virtually complete follow-up. Prescription data are not subject to recall bias, and statins are in Denmark available only by prescription. Large observational studies with long-term follow-up are valuable when investigating the influence of statin use on CBC risk, given the difficulties to implement a randomised trial among breast cancer patients with sufficient power to detect rare and long-term adverse outcomes, such as CBCs.^37^

Our study also had some limitations. We had no information on compliance to statins and other drug use. We attempted to minimise exposure misclassification by defining drug use as at least two prescriptions filled on separate dates. In addition, adherence to statins in Denmark has been reported to be high (>84%).^38^ Statin users were more likely to have shorter education and comorbidities than non-users, which do not support that the overall group of statin users in our study had a heathier lifestyle. Nevertheless, we cannot rule out that long-term low intensity statin use might reflect adherence to a healthier lifestyle. In all instances, the analyses were adjusted for education and comorbidity that are indicators of lifestyle.^39–42^ Our results suggested no major difference in overall risk estimates according to timing of statin use indicating that prevalent user bias may not be a major concern. Further analyses on patterns of statin use and ER status restricted to ‘new users’ are not likely to provide additional information on the issue of prevalent user bias due to limited statistical precision. Patients with recurrence following primary breast cancer may be more unlikely to be registered with a new primary tumour in the contralateral breast than those without recurrence. Since statin users tend to have fewer recurrences, this may have biased the association toward the null. In observational studies of statins and cancer risk reverse causality might impact results if patients undiagnosed with cancer experience weight loss and improved lipid profiles and consequently do not use statins. However, this is unlikely to have affected our results to any great extent because general or non-breast symptoms are uncommon in patients with primary breast cancer.^43^ In addition, we censored patients with distant metastases at CBC diagnosis and other cancer diagnoses for whom disease symptoms potentially could have affected their use of statins. Finally, we cannot exclude the possibility of important residual confounding from unmeasured variables associated with both statin use and CBC risk.

In conclusion, we found some support of a reduced risk of CBC associated with statin use among breast cancer patients. Further studies are required to clarify the equivocal findings according to pattern of statin use, notably for long-term use, and to determine if statin use could be particularly useful for women with breast cancer of the ER-negative phenotype.

## Electronic supplementary material

Supplemental material
