# Supplementary material for: Statin use and risk of contralateral breast cancer: a nationwide cohort study
Source: Br J Cancer. 2018 Oct 24;119(10):1297–305. doi: 10.1038/s41416-018-0252-1 (PMC6251024; doi:10.1038/s41416-018-0252-1)
Supplement: Supplementary file 1 — Supplemental material [file 41416_2018_252_MOESM1_ESM.docx]

**Supplementary Material**

**Statin use and risk of contralateral breast cancer: a nationwide cohort study**

**Authors:**

Rikke Langballe^1^, Deirdre Cronin-Fenton^2^, Christian Delendorff^1^, Maj-Britt Jensen^3^, Bent Ejlertsen^3+4^, Michael Andersson^4^, Søren Friis^1,5^ and Lene Mellemkjær^1^

**Affiliations:**

^1^Danish Cancer Society Research Center, Copenhagen, Denmark, ^2^Department of Clinical Epidemiology, Aarhus University Hospital, Denmark, ^3^Danish Breast Cancer Group, Rigshospitalet, Copenhagen, Denmark’ ^4^Department of Oncology at Rigshospitalet, Copenhagen, Denmark, ^5^Department of Public Health, Copenhagen University, Denmark.

**Corresponding author:**

Lene Mellemkjær, Danish Cancer Society Research Center, Strandboulevarden 49, 2100 Copenhagen, Denmark, telephone: +45 3525 7612, e-mail: lene@cancer.dk

**Appendix Table 1. Codes used to assess statin exposure, confounding and censoring variables**

|  | **Codes** |
| --- | --- |
| **Statin exposure** |  |
| Lipophilic statins |  |
| Simvastatin | ATC: C10AA01 |
| Simvastatin + ezetimibe | ATC: C10BA02 |
| Atorvastatin | ATC: C10AA05 |
| Atorvastatin+ ezetimibe | ATC: C10BA05 |
| Lovastatin | ATC: C10AA02 |
| Fluvastatin | ATC: C10AA04 |
| Cerivastatin | ATC: C10AA06 |
| Hydrophilic statins |  |
| Pravastatin | ATC: C10AA03 |
| Rosuvastatin | ATC: C10AA07 |
| **Confounding variables** |  |
| Aspirin | ATC: B01AC06, N02BA01, N02BA51 |
| Bisphosphonates | ATC: M05BA, M05BB |
| Metformin | ATC: A10BA02 |
| Digoxin | ATC: C01AA05 |
| Hormone replacement therapy | ATC: G03C, G03D, G03F, G03HB01 |
| **Comorbidities** |  |
| Alcohol related diseases | ICD-8: 291, 303, 577.19, 979, 980  ICD-10: F10, G31.2, G62.1, G72.1, I42.6, K29.2, K70, K86.0, R78.0, T51, Z72.1 |
| Tobacco related diseases | ICD-8: 491­-493  ICD-10: J41-46 |
| Diabetes mellitus | ICD-8: 249, 250  ICD-10: E10, E11 |
| **Censoring variable** |  |
| Mastectomy of the contralateral breast | Surgical code^a^: KHAC combined with the laterality code TUL1/2 |

^a^Nordic Classification of Surgical Procedures accessed from <http://www.medinfo.dk/sks/brows.php>

Abbreviations: ATC, Anatomical Therapeutic Chemical, ICD, International Classification of Diseases.

**Appendix Box 1. Description of registries used in the study**

The *Danish Breast Cancer Cooperative Group* (DBCG) database became nationwide in 1977 and includes almost complete registration of invasive breast cancer since 1996 with detailed clinical information on the vast majority of women^1^. The information include tumor characteristics and treatment reported on standardized forms from hospital departments. Patients are allocated to evidence-based treatment protocols according to tumor characteristics and age. Over time, more tumor characteristics and lower age were added resulting in increasing number of patients receiving radiotherapy and systemic treatment.

The *Danish Cancer Registry* offers accurate and almost complete records of incident cancer in Denmark since 1943^2,3^. Data consists of information from hospitals, pathology departments, physicians and death certificates. The early period of the cancer registry (1943–1978) coded cancer diagnoses according to the International Classification of Diseases version 7 (ICD-7), and during 1978–2003 these codes were systematically converted to ICD-10 codes. After 2004, all cancer diagnoses are reported in ICD-10 codes.

The *Danish National Prescription Registry* contains records for drug prescriptions in Denmark since 1995^4,5^. The records include the type and quantity of drug dispensed, and the date of dispensing. The indications for prescribing and dosing schedules are not available, and no information is available on drugs dispensed at hospital level. Drugs are categorized according to the Anatomical Therapeutic Chemical (ATC) Classification System, a hierarchical classification system developed by the WHO^6^. For each prescription, the quantity of drug is given as the strength and number of pharmaceutical units (e.g., tablets), as well as the number of defined daily doses (DDDs). The DDD for a drug represents the typical daily dose required by an adult when the drug is used for its main indication (e.g., 30 mg for simvastatin)^6^.

The *Danish Pathology Register* ^7^ has collected data from all departments of pathology since 1997 based on a Danish version of the Systematized Nomenclature of Medicine (SNOMED)^8^.

The *Danish National Patient Register* contains detailed information on diagnoses and procedures for all somatic hospital admissions in Denmark since 1977 and on all ambulatory (out-patient) hospital contacts and psychiatric admissions since 1995^9^. Diagnoses are coded according to ICD-8 (1977–1993) and ICD-10 (>1994) while procedures are registered as the Danish Classification of Surgical Procedures and Therapies^10^ (1977–1995) and the Nordic Classification of Surgical Procedures (>1996)^11^.

The *Danish Civil Registration System*^12^ was instituted in 1968 and since then, all citizens in Denmark have been assigned a unique personal identification number used for accurate linkage between the nationwide registries. Individual information on vital status, address and emigration to and from Denmark is continuously updated.

*Statistics Denmark*^13^ is a national institution that collects, administers and publishes statistical information regarding the Danish society, such as information on highest attained education of the Danish population.

**Appendix Table 2. Hazard ratios (HRs) of contralateral breast cancer (CBC) and 95% confidence intervals (CIs) associated with post-diagnostic lipophilic statin use only according to estrogen receptor (ER) status of first breast cancer (BC) diagnosis and CBC among 52 723 breast cancer patients during 1996–2012 in Denmark**

|  | **Person-years** | **N** | **Number**  **of CBCs** | **Age-adjusted**  **model** | |  | **Fully adjusted**  **model^a^** | |
| --- | --- | --- | --- | --- | --- | --- | --- | --- |
|  |  |  |  | **HR** | **95% CI** |  | **HR** | **95% CI** |
| **ER status of first breast cancer** | |  |  |  |  |  |  |  |
| **Positive** |  |  |  |  |  |  |  |  |
| Non-use | 208 219 | 41 593 | 885 | 1 | Reference |  | 1 | Reference |
| Ever statin use | 31 139 | 8 773 | 124 | 0.86 | 0.71–1.04 |  | 0.91 | 0.74–1.12 |
| **Negative** |  |  |  |  |  |  |  |  |
| Non-use | 49 257 | 9 824 | 274 | 1 | Reference |  | 1 | Reference |
| Ever statin use | 6 836 | 1 787 | 27 | 0.71 | 0.47–1.05 |  | 0.73 | 0.49–1.10 |
| **Unknown** |  |  |  |  |  |  |  |  |
| Non-use | 9 313 | 1 306 | 45 | 1 | Reference |  | 1 | Reference |
| Ever statin use | 1 118 | 263 | 6 | 0.16 | 0.07–0.40 |  | 1.18 | 0.07–0.43 |
| **ER status of contralateral breast cancer^b^** | | |  |  |  |  |  |  |
| **Positive^c^** | | |  |  |  |  |  |  |
| Non-use | 266 790 | 52 723 | 818 | 1 | Reference |  | 1 | Reference |
| Ever statin use | 39 093 | 10 823 | 118 | 0.83 | 0.68–1.02 |  | 0.84 | 0.68–1.05 |
|  |  |  |  |  |  |  |  |  |
| **Negative^d^** |  |  |  |  |  |  |  |  |
| Non-use | 266 790 | 52 723 | 214 | 1 | Reference |  | 1 | Reference |
| Ever statin use | 39 093 | 10 823 | 25 | 0.98 | 0.64–1.50 |  | 0.96 | 0.60–1.55 |
| **ER status of first and second breast cancer** | | |  |  |  |  |  |  |
| **ER-positive first BC and ER-positive CBC^c^** |  |  |  |  |  |  |  |  |
| Non-use | 208 219 | 41 593 | 652 | 1 | Reference |  | 1 | Reference |
| Ever statin use | 31 139 | 8 773 | 95 | 0.84 | 0.68–1.05 |  | 0.89 | 0.70–1.13 |
| **ER-positive first BC and ER-negative CBC^d^** |  |  |  |  |  |  |  |  |
| Non-user | 208 219 | 41 593 | 120 | 1 | Reference |  | 1 | Reference |
| Ever statin use | 31 139 | 8 773 | 17 | 0.95 | 0.57–1.61 |  | 0.98 | 0.54–1.75 |
| **ER-Negative first BC and ER-positive CBC^c^** |  |  |  |  |  |  |  |  |
| Non-use | 49 257 | 9 824 | 142 | 1 | Reference |  | 1 | Reference |
| Ever statin use | 6 837 | 1 787 | 20 | 0.78 | 0.48–1.26 |  | 0.68 | 0.40–1.17 |
| **ER-Negative first BC and ER-negative CBC^d^** |  |  |  |  |  |  |  |  |
| Non-use | 49 257 | 9 824 | 86 | 1 | Reference |  | 1 | Reference |
| Ever statin use | 6 837 | 1 787 | 6 | 0.81 | 0.34–1.90 |  | 0.85 | 0.34–2.17 |

**^a^** Adjusted for age at first breast cancer, calendar-period at first breast cancer (1996–2000/2001–2004/2005–2008/2009–2012), lobular histology of first breast cancer (yes/no), treatment for first breast cancer (endocrine treatment only, chemotherapy only, radiation treatment only, endocrine treatment + chemotherapy, endocrine treatment + radiation treatment, chemotherapy + radiation treatment, endocrine treatment + chemotherapy + radiation treatment, no treatment and unknown treatment), pre-diagnosis exposure to hormone-replacement therapy (yes/no), time-dependent post-diagnostic exposure to aspirin, bisphosphonates, metformin and digoxin, alcohol-related conditions (yes/no), tobacco-related conditions (yes/no), diabetes mellitus (yes/no), educational level at first breast cancer diagnosis (short, medium, higher, unknown).

^b^ We have not estimated HR for unknown ER status of the CBC.

^c^In these sub analyses, ER-positive contralateral breast cancer was the outcome of interest and ER-negative and unknown ER status was censuring variables plus all the censuring variables used in all other analyses.

^d^In these sub analyses, ER-negative contralateral breast cancer was the outcome of interest and ER-positive and unknown ER status was censuring variables plus all the censuring variables used in all other analyses.

**Supplementary references**

1. Christiansen P, Ejlertsen B, Jensen MB, Mouridsen H. Danish Breast Cancer Cooperative Group. *Clin Epidemiol* 2016; **8**: 445-449.

2. Gjerstorff ML. The Danish Cancer Registry. *ScandJPublic Health* 2011; **39**: 42-45.

3. Storm HH, Michelsen EV, Clemmensen IH, Pihl J. The Danish Cancer Registry--history, content, quality and use. *DanMedBull* 1997; **44**: 535-539.

4. Kildemoes HW, Sorensen HT, Hallas J. The Danish National Prescription Registry. *Scandinavian journal of public health* 2011; **39**: 38-41.

5. Pottegard A, Schmidt SAJ, Wallach-Kildemoes H, Sorensen HT, Hallas J, Schmidt M. Data Resource Profile: The Danish National Prescription Registry. *Int J Epidemiol* 2017; **46**: 798-798.

6. WHO. Guidelines for ATC calssification and DDD assignment. In. *World Health Organization Collaborating Centre for Drug Statistics Methodology* 2017.

7. Bjerregaard B, Larsen OB. The Danish Pathology Register. *ScandJPublic Health* 2011; **39**: 72-74.

8. National Board of Health. *Kodebog for patologisk-anatomiske undersøgelser (Code book for pathologic and anatomical examinations)*, 3rd ed. National Board of Health: Denmark.

9. Schmidt M, Schmidt SA, Sandegaard JL, Ehrenstein V, Pedersen L, Sorensen HT. The Danish National Patient Registry: a review of content, data quality, and research potential. *Clin Epidemiol* 2015; **7**: 449-490.

10. National Board of Health. *Operations- og behandlingsklassifikation (Danish Classification of Surgical Procedures and Therapies)*, 1st, 2nd, 3rd and 4th ed. Schultz Grafisk A/S: Copenhagen, 1973, 1980, and 1988.

11. Nordic Classification of Surgical Procedures. <http://www.medinfo.dk/sks/brows.php>.

12. Pedersen CB. The Danish Civil Registration System. *Scandinavian journal of public health* 2011; **39**: 22-25.

13. Jensen VM, Rasmussen AW. Danish Education Registers. *Scandinavian journal of public health* 2011; **39**: 91-94.
